# Sporadic Creutzfeldt-Jakob disease VM1: phenotypic and molecular characterization of a novel subtype of human prion disease

**DOI:** 10.1186/s40478-022-01415-7

**Published:** 2022-08-17

**Authors:** Ellen Gelpi, Simone Baiardi, Carlos Nos, Sofia Dellavalle, Iban Aldecoa, Raquel Ruiz-Garcia, Lourdes Ispierto, Domingo Escudero, Virgina Casado, Elena Barranco, Anuncia Boltes, Laura Molina-Porcel, Nuria Bargalló, Marcello Rossi, Angela Mammana, Dorina Tiple, Luana Vaianella, Elisabeth Stoegmann, Ingrid Simonitsch-Klupp, Gregor Kasprian, Sigrid Klotz, Romana Höftberger, Herbert Budka, Gabor G. Kovacs, Isidre Ferrer, Sabina Capellari, Raquel Sanchez-Valle, Piero Parchi

**Affiliations:** 1 Division of Neuropathology and Neurochemistry, Department of Neurology, Medical University of Vienna and Austrian Reference Center for Human Prion Diseases (ÖRPE), AKH Leitstelle 4J, Waehringer Guertel 18-20, 1090 Vienna, Austria; 2 Neurological Tissue Bank of the Biobank-Hospital Clinic-IDIBAPS, Barcelona, Spain; 3 IRCCS Istituto delle Scienze Neurologiche di Bologna, Via Altura 1/8, 40139 Bologna, Italy; 4 Department of Biomedical and Neuromotor Sciences (DIBINEM), University of Bologna, Bologna, Italy; 5 General Subdirectorate of Surveillance and Response to Emergencies in Public Health, Department of Public Health in Catalonia, Barcelona, Spain; 6 Department of Pathology, Center for Biomedical Diagnosis, Hospital Clinic de Barcelona, University of Barcelona, Barcelona, Spain; 7 Department of Immunology, Center for Biomedical Diagnosis, Hospital Clinic de Barcelona, Barcelona, Spain; 8 Cognitive and Movement Disorders Unit, Hospital Germans Trias I Pujol de Badalona, Barcelona, Spain; 9 Neurology Department, Hospital de Mataró, Barcelona, Spain; 10 Department of Geriatrics, Hospital General de Granollers, Barcelona, Spain; 11 Department of Neurology, Hospital General de Granollers, Barcelona, Spain; 12 Neurology Department, Alzheimer Disease and Other Cognitive Disorders Unit, Hospital Clinic de Barcelona, Barcelona, Spain; 13 Radiology Department, Image Diagnosis Center, Hospital Clínic de Barcelona, Spain and Magnetic Resonance Image Core Facility of IDIBAPS, Barcelona, Spain; 14 Department of Neuroscience, Istituto Superiore di Sanità, 00161 Rome, Italy; 15 Department of Neurology, Medical University of Vienna, Vienna, Austria; 16 Department of Pathology, Medical University of Vienna, Vienna, Austria; 17 Department of Biomedical Imaging and Image-Guided Therapy, Medical University of Vienna, Vienna, Austria; 18 Tanz Centre for Research in Neurodegenerative Disease, University of Toronto, Toronto, ON Canada; 19 Department of Laboratory Medicine and Pathobiology and Department of Medicine, University of Toronto, Toronto, ON Canada; 20 Laboratory Medicine Program & Krembil Brain Institute, University Health Network, Toronto, ON Canada; 21 Department of Pathology and Experimental Therapeutics, University of BarcelonaBellvitge University Hospital-IDIBELLCIBERNED, Barcelona, Spain

**Keywords:** CJD, PrP, Prion disease, Histotype, Classification, *PRNP*, Prion strains, Codon 129

## Abstract

The methionine (M)—valine (V) polymorphic codon 129 of the prion protein gene (*PRNP*) plays a central role in both susceptibility and phenotypic expression of sporadic Creutzfeldt-Jakob diseases (sCJD). Experimental transmissions of sCJD in humanized transgenic mice led to the isolation of five prion strains, named M1, M2C, M2T, V2, and V1, based on two major conformations of the pathological prion protein (PrP^Sc^, type 1 and type 2), and the codon 129 genotype determining susceptibility and propagation efficiency. While the most frequent sCJD strains have been described in codon 129 homozygosis (MM1, MM2C, VV2) and heterozygosis (MV1, MV2K, and MV2C), the V1 strain has only been found in patients carrying VV. We identified six sCJD cases, 4 in Catalonia and 2 in Italy, carrying MV at *PRNP* codon 129 in combination with PrP^Sc^ type 1 and a new clinical and neuropathological profile reminiscent of the VV1 sCJD subtype rather than typical MM1/MV1. All patients had a relatively long duration (mean of 20.5 vs. 3.5 months of MM1/MV1 patients) and lacked electroencephalographic periodic sharp-wave complexes at diagnosis. Distinctive histopathological features included the spongiform change with vacuoles of larger size than those seen in sCJD MM1/MV1, the lesion profile with prominent cortical and striatal involvement, and the pattern of PrP^Sc^ deposition characterized by a dissociation between florid spongiform change and mild synaptic deposits associated with coarse, patch-like deposits in the cerebellar molecular layer. Western blot analysis of brain homogenates revealed a PrP^Sc^ type 1 profile with physicochemical properties reminiscent of the type 1 protein linked to the VV1 sCJD subtype. In summary, we have identified a new subtype of sCJD with distinctive clinicopathological features significantly overlapping with those of the VV1 subtype, possibly representing the missing evidence of V1 sCJD strain propagation in the 129MV host genotype.

**Supplementary Information:**

The online version contains supplementary material available at 10.1186/s40478-022-01415-7.

## Introduction

Increasing evidence indicates that heterogeneous protein amyloid aggregates differing in structural and physicochemical properties, commonly named strains, sustain the molecular basis of phenotypic heterogeneity in prion disease and other neurodegenerative disorders [1, 5, 26, 29, 30]. After injection into syngeneic hosts, prion strains cause diseases with distinct characteristics, such as incubation period, regional severity of neuropathological changes (i.e., the lesion profile), and pattern of tissue deposition of misfolded (scrapie) prion protein (PrP^Sc^) [1, 5]. Moreover, strain-specific PrP^Sc^ physicochemical properties, such as the size of its protease-resistant core fragment, often propagate after transmission [6, 31]. Finally, changes in the host prion protein gene (*PRNP*) sequence may also affect the phenotypic expression of a given strain [5].

Reflecting this evidence, the current histo-molecular classification of sporadic Creutzfeldt-Jakob disease (sCJD), the most common human prion disease, largely relies on the association between a host polymorphism at *PRNP* codon 129 (coding for methionine/M or valine/V), and two distinct types of the disease-related prion protein (PrP^Sc^ type 1, and type 2) differing in the molecular mass of their protease-resistant fragment [21, 25]. The combinations of the two variables specify six molecular groups, MM1, MV1, VV1, MM2, MV2, and VV2, which primarily explain the clinical and neuropathological phenotypic heterogeneity of sCJD [23]. Given that two molecular combinations are associated with two distinct clinicopathological subtypes rather than one, the classification also refers to "histopathological" hallmarks. Accordingly, the MM2 group includes a "cortical" (MM2C) and a "thalamic" variant (MM2T), and the MV2 group consists of a prevalent subtype with cerebellar kuru-type amyloid plaques (MV2K subtype) and a rare variant phenotypically indistinguishable from the MM2C group (MV2C subtype). Intriguingly, the classification also applies to the prevalent genetic forms of CJD (gCJD), where the histotypes mainly depend on the codon 129 polymorphism/PrP^Sc^ types 1 and 2 combinations rather than the mutation per se [4].

Experimental transmission of the six sCJD subtypes led to the isolation of five prion strains, named M1, M2C, M2T, V2, and V1, based on both the codon 129 genotype determining the preferential susceptibility (i.e., transmission rate and incubation time), and the distinctive phenotypic features. Each subtype maintained unique features in the recipient animals, except for the VV2 and MV2K, which converged in one strain, named V2, because of the preferential conversion of the PrP with V at residue 129 [3, 7, 22]. Interestingly, the most frequent sCJD strains have been described both in codon 129 homozygosis (M1 in MM1, M2C in MM2C, V2 in VV2) and heterozygosis (M1 in MV1, M2C in MV2C, and V2 in MV2K) [24]. As one significant exception, the V1 strain has only been found in homozygotes at codon 129, leading to the assumption that all cases showing the MV1 combination are linked to the M1 strain.

Histotype-based characterization of the postmortem human brain has proved advantageous for identifying the sCJD and gCJD subtypes and the associated strains [4, 20]. Indeed, most, if not all, CJD subtypes show histopathological hallmarks that distinguish them more accurately than the molecular characterization based on PrP^Sc^ typing and *PRNP* sequencing alone. Moreover, the histopathological approach is remarkably accurate in identifying mixed phenotypes that combine the presence of PrP^Sc^ types 1 and 2 in the same brain, as neuropathological alterations may be very focal and region-dependent [24]. The same approach also distinguishes strains sharing the same PrP^Sc^ profile at the western blot (e.g., MM2T + 2C or MV2K + 2C). Finally, histotyping identifies atypical phenotypes that do not match the conventional patterns and detects discrepancies between the patient's clinical phenotype, genotype, and expected neuropathology. As confirmation, histotyping represented the critical approach that led to the discovery of variably protease-resistant prionopathy, the last identified sporadic human prion disease [10].

Here we describe the clinical, neuropathological, and molecular features of six patients from Spain and Italy affected by a previously unreported disease subtype, possibly representing the missing V1 strain in host 129 heterozygosis MV. We will refer to this condition as sCJD VM1 subtype. We have identified these cases primarily through a histotyping approach within prion disease national surveillance programs, revealing a distinct signature from the previously established sCJD subtypes.

## Materials and methods

The cases were identified at the Neurological Tissue Bank of the Hospital Clinic de Barcelona-IDIBAPS Biobank, Barcelona, Spain (n = 4), and at the Laboratory of Neuropathology (LabNP) of the Institute of Neurological Science of Bologna (ISNB), Italy (n = 2). The 6 cases were detected within a series of 1100 CJD brains examined at the two centers mentioned above and at the Division of Neuropathology and Neurochemistry of the Medical University of Vienna (MUW); 150 at IDIBAPS Barcelona (60% MM, 15% MV and 25% VV), 300 at MUW Vienna (60% MM, 23% MV, 18% VV), and 650 at LabNP of ISNB (71% MM, 15% MV, 14% VV). Participants were collected through the national CJD surveillance systems as all the three Institutions act as the local reference center for the neuropathological assessment of human prion diseases in their respective countries.

### CJD histotyping

CJD histotyping was performed according to consensus international guidelines at each center [20]. Formalin-fixed, paraffin-embedded, and formic acid pretreated tissue sections from at least eight brain regions, including frontal, temporal, and occipital neocortices, hippocampus with parahippocampal gyrus, anterior striatum, medial and lateral thalamus, midbrain, and cerebellum were stained with hematoxylin–eosin and PrP-immunohistochemistry (Merck Millipore, Milan, Italy, clone 3F4, epitope aa 109-112, dilution 1:300; and,  Caymann Chemical, Ann Arbor, MI, USA, clone 12F10, epitope aa 142–160, dilution 1:1,000) [14].

In each section, we assessed the degree (absent, −/mild, +/moderate, ++ /severe, +++/status spongiosus ++++) and distribution (deep laminar/transcortical/confluent) of spongiform change and the type (synaptic/deep perineuronal/patchy-perivacuolar/plaque-like/PrP amyloid plaques) and intensity (absent/mild/moderate/intense) of pathological PrP deposits. Immunohistochemistry was performed in a Dako autostainer plus in Barcelona and Vienna, whereas it was carried out manually in Bologna, as previously described [4].

As a part of the routine neuropathological examination and to assess the presence of concomitant pathologies, all brains were stained with anti-Tau (Thermo Scientific, Rockford, IL, USA, clone AT8, dilution 1:200), anti-β-Amyloid (Dako, Glostrup, Denmark, clone 6F/3D, dilution 1:400), anti-alpha-synuclein (Novocastra, Newcastle UK, clone KM51, dilution 1:500 or clone 5G4, Roboscreen, Leipzig, Germany, dilution 1:4000), anti-p62 (BDK Transduction Laboratories, Franklin Lakes, NJ, USA, clone 3/p62 lck ligand, dilution 1:500), and anti-TDP43 (Abnova, clone 2E2-D3, dilution 1:500) or anti-pTDP43 (Cosmo Bio, Tokyo, Japan, clone pD409/410, dilution 1:2000) antibodies in selected brain areas.

Clinical data were retrospectively retrieved from the brain bank files and the treating neurologists.

### Molecular genetic studies

We analyzed the *PRNP* open reading frame according to a previously published protocol [4]. In brief, we amplified genomic DNA extracted from frozen brain tissue (n = 2) or peripheral white blood cells (n = 4) by polymerase chain reaction using specific primers. In each patient, we then determined the genotype at codon 129 and ruled out pathogenic mutation by direct sequencing.

### Western blotting and PrP^Sc^ typing

Frozen brain samples (50–100 mg) of gray matter from the frontal cortex and cerebellum (Spanish cases) or twelve different brain regions, including the frontal, temporal, parietal, and occipital cortices, striatum, hippocampus, thalamus, midbrain, pons, medulla, and cerebellum (vermis and hemisphere) (Italian cases) were homogenized (10%, w/v) in LB100, a lysis buffer with high buffer capacity (100 mM Tris, 100 mM NaCl, 10 mM EDTA, 0,5% Nonidet P-40, 0,5% sodium deoxycholate) prepared at two different pHs, 6.9 and 8.0.

Aliquots were treated for 1 h at 37 °C with 200 mg/ml of proteinase K (PK) (with a PK specific activity of 20 U/mg). Appropriate positive controls (i.e., MM1, MV1, and VV2 sCJD brain samples) were PK-digested in parallel. PK treated samples were then mixed with SDS-PAGE sample buffer and boiled at 100 °C for 6 min before gel loading. Proteins were resolved in 13% polyacrylamide gels using either a medium-sized or a long gel electrophoresis apparatus (both from Bio-Rad Laboratories S.r.l., Mlan, Italy) and transferred to Immobilon-P membranes (Millipore Corp., Billerica, MA). After blocking in 10% nonfat milk in Tween-Tris-buffered saline, membranes were incubated overnight with the monoclonal antibody (mAb) 3F4 (Signet Laboratories, Dedham, MA, working dilution, 1:30000), which maps on residues 109–112 or with the antiserum 2301 (working dilution 1:4000) which recognizes a C-terminal epitope (residues 220–231). After four washing steps in Tween-Tris-buffered saline, membranes were incubated for 1 h with an anti-mouse or an anti-rabbit secondary antibody conjugated to horseradish peroxidase (HRP; working dilution, 1:4000 or 1:3000; GE Healthcare) and rewashed four times in Tween-Tris-buffered saline. The immunoreactive signal was detected by enhanced chemiluminescence (Immobilon Western Chemiluminescent HRP substrate; Merck Millipore, Milan, Italy) on a LAS 4000 camera (Fujifilm Corp., Tokyo, Japan). Western blot signals were measured by densitometry using the software AIDA (Image Data Analyzer v.4.15; Raytest Isotopenmessgeraete GmbH, Straubenhardt, Germany).

## Results

We have identified six patients, four in Spain and two in Italy, affected by a novel sCJD subtype with distinctive histopathological and molecular features.

### ***Common clinical features (for details, see Table ******1******)***

**Table 1:** Basic demographic and clinical features

Case-country	Sex	AAO (yrs.)	DD (mos.)	Symptoms at onset	Symptoms during disease course	EEG	DW-MRI	CSF analyses	
								14–3-3 protein	RT-QuIC
#1-ES	F	78	10	Rapidly progressive dementia	Gait ataxia, myoclonus, mixed rest and postural tremor, limb apraxia	Unspecific	Cortical hyperintensities	+	n.p
#2-ES	M	78	24	Cognitive decline	Hallucinations, emotional lability, parkinsonism, myoclonus	n.p	n.p	+	n.p
#3-ES	M	73	18	Progressive hemi-hypoestesia, gait disorder, parkinsonism, myoclonus	Rapidly progressive cognitive decline, executive dysfunction, emotional lability	Unspecific diffuse slowing	Cortical hyperintensities	+	n.p
#4-ES	M	82	21	Dizziness, gait disturbance, subacute hemiparesis, focal seizures	Bradykinesia, gait apraxia, dysarthria, rapidly progressive cognitive decline, apathy, myoclonus, akinetic mutism	Focal slowing	Cortical hyperintensities	+	+ (PQ)
#5-IT	M	70	28	Rapidly progressive parkinsonism	Cerebellar ataxia, memory loss, visual hallucination, delirium, akinetic mutism	Unspecific diffuse slowing	Cortical hyperintensities	+	+ (both PQ and IQ)
#6-IT	M	67	22	Excessive daytime sleepiness, blurred vision, dizziness	Rapidly progressive behavioral alteration and cognitive decline, cerebellar ataxia, myoclonus, akinetic mutism	Unspecific diffuse slowing	Cortical hyperintensities	_	+ (PQ); − (IQ)

*ES* Spain, *IT* Italy, *F* female, *M* male, *AAO* age at disease onset, *yrs* years, *DD* disease duration, *mos*. months, *EEG* electroencephalography, *DW-MRI* diffusion-weighted magnetic resonance imaging, + positive, − negative, *n.p* not performed, *CSF* cerebrospinal fluid, *RT-QuIC* real-time quaking-induced conversion, *PQ* previous QuIC protocol (Ha rPrP23-231), *IQ* improved QuIC protocol (Ha rPrP90-231)

Five patients were males and one female. The age at disease onset ranged from 67 to 82 years, and the clinical disease duration from 10 to 24 months (mean 20.5). Most patients presented with symptoms suggestive of prion disease; as the only exception, patient #5 was initially diagnosed with Parkinson's disease but later suspected of having CJD because of the rapid progression. The symptoms were rapidly progressive and combined cognitive impairment, behavioral alterations, emotional lability, and executive dysfunction associated with various motor symptoms and myoclonus. Most patients developed akinetic mutism and were bedridden in less than one year. Electroencephalographic recordings (EEG) showed non-specific changes, and none of the patients showed periodic sharp wave complexes at diagnostic work-up. The protein 14–3-3 assay in cerebrospinal fluid (CSF) was positive in 5 of the 6 patients. The CSF real-time quaking-induced conversion assay was performed retrospectively in three patients and gave a positive outcome. Brain MRI showed cortical hyperintensities in all five patients examined (Table 1, Additional file 1: Fig. S1).

**Fig. 1:**
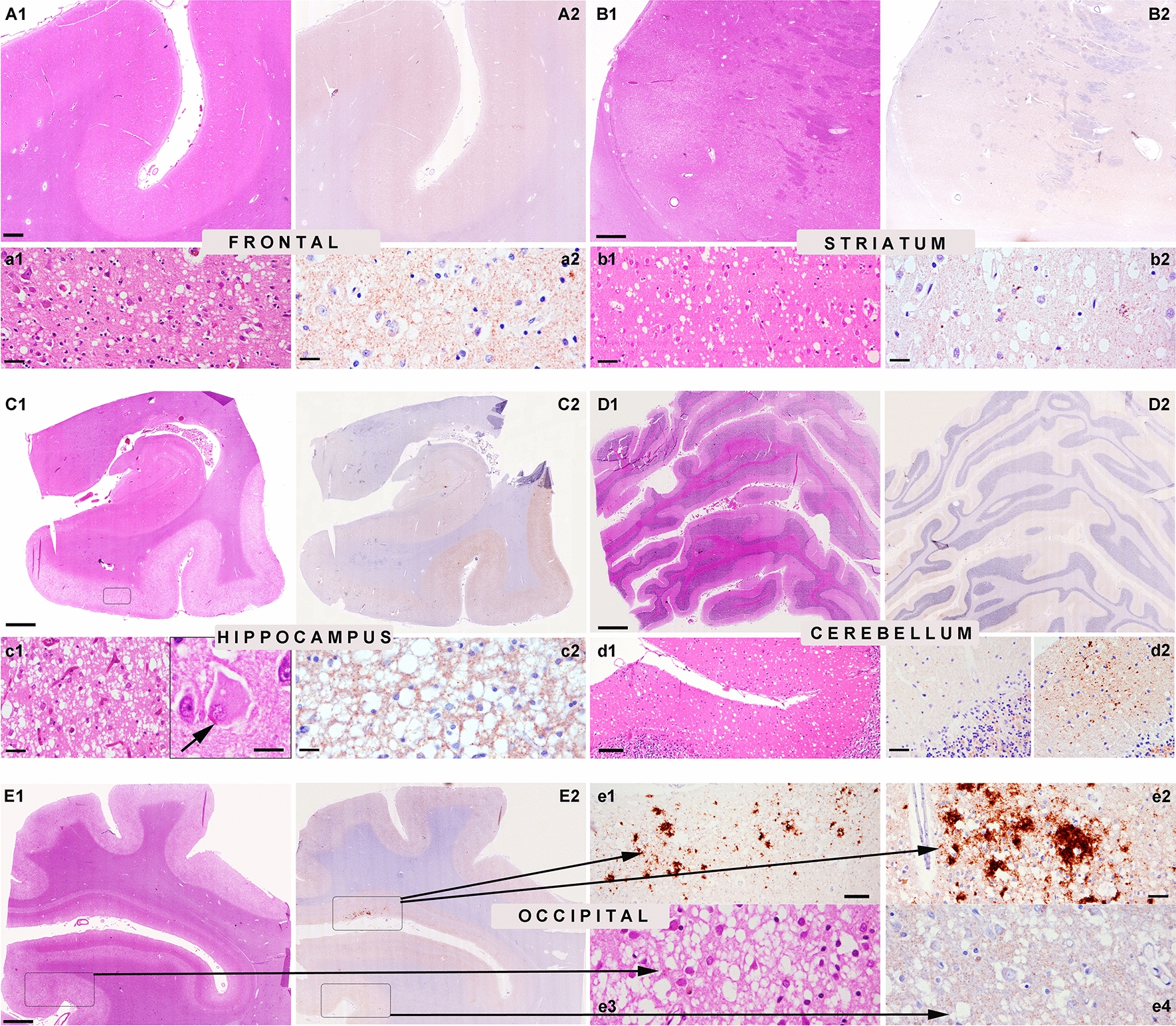
Characteristic histopathological features in the reported sCJD cases. Prominent spongiform change is observed in HE-stained sections in the frontal cortex (**A1**, **a1** higher magnification), striatum (**B1**, **b1** higher magnification), parahippocampal region (**C1**, **c1** higher magnification), and occipital cortex (**E1**, **e3** higher magnification). Ballooned neurons are observed in severely affected brain areas (**c1**, arrow inset). There is a striking dissociation between the marked spongiform change and the very faint deposition of pathological prion protein (PrP) by immunohistochemistry (anti-PrP antibody 12F10) in the frontal cortex (**A2**, **a2** higher magnification), striatum (**B2**, **b2** higher magnification), parahippocampal region (**C2**, **c2** higher magnification) and in most areas of the occipital cortex (**E2**, **e4** higher magnification). There are focal areas (square in **E2**) with coarse patchy-like PrP deposits (**e1**, **e2**). In the cerebellum (**D1**), spongiform change is focally prominent in the molecular layer (**d1**), and also here, there is a dissociation between the rather severe spongiform change and the mild PrP deposits (**D2**, **d2** left, higher magnification). Focally, deposits appear coarser and patchy (**d2**, right panel). *Scale bars: 10 μm: ****c1**** inset, ****a2****, ****b2****, ****c2****, ****e3****, ****e4****; 20 μm: ****a1****, ****b1****, ****c1****, ****d2****, ****e2****; 50 μm: ****d1****, ****e1****; 500 μm: ****A1****, ****A2****, ****B1****, ****B2****; 1,2 mm: ****C1****, ****C2****, ****D1****, ****D2****, ****E1****, ****E2****. Panels ****A1****, ****A2****, ****B1****, ****B2****, ****C1****, ****C2****, ****E1****, ****E2**** correspond to patient 1 and panels ****D1****, ****D2**** to patient 2*

### ***Common neuropathological features (for details, see Table ******2****** and Fig. ******1******)***

**Table 2:** Neuropathologic findings in the six atypical sCJD cases

	Case					
	#1	#2	#3	#4	#5	#6
*Frontal*						
SP	++	++/+++	+++	+++/++++	++/+++	++
PrP	Faint syn	Faint syn	Faint syn	Faint syn	Faint syn	Faint syn
*Temporal*						
SP	++/+++	++/+++	+++	+++	++/+++	++/+++
PrP	Faint syn	Faint syn	Faint syn	Faint syn	Faint syn	Negative
*Parietal*						
SP	++	++	++/+++	+++	++	++/+++
PrP	Faint syn	Faint syn	Faint syn	Faint syn	Faint syn	Faint syn
*Occipital*						
SP	+++/++++	+++	+++/++++	+++	++	++/+++
PrP	Faint syn, patchy*	Faint syn	Faint syn	Faint syn, patchy*	Faint syn	Faint syn
*Striatum*						
SP	++/+++	++	++	+++	+	+
PrP	Faint syn	Faint syn	Faint syn	Faint syn	Faint syn	Faint syn
*Thalamus*						
SP	+	+	+	+	+	+
PrP	Negative	Faint syn	Faint syn	Faint syn	Negative	Negative
*Hippocampus*						
SP	+	−	−	−/+	−	−
PrP	Negative	Negative	Negative	Negative	Negative	Negative
*Parahippocampus*						
SP	+++/++++	++/+++	+++/++++	+++/++++	++/+++	++/+++
PrP	Faint syn	Faint syn	Faint syn	Faint syn	Faint syn	Faint syn
*Brainstem*						
SP	−/+	−/+	−/+	−/+	+/++	−
PrP	Negative	Negative	Negative	Negative	Plaque-like	Negative
*Cerebellum*						
SP	++	+	++	++	+	+/++
PrP	Syn, patchy*	Syn, patchy*	Syn, patchy*	Syn, patchy*	Syn, patchy*	Syn, patchy*
Balloon neurons	Yes	Yes	Isolated	Yes	Isolated	No
Copathologies	AgD III, ARP: A1,B1,C1 (“possible” PART)	AgD III, ARP: A2,B1,C2	ARP: A1,B1,C1 (“possible” PART), mild CAA	LB limbic, mod-prominent CAA, “definite” PART (Braak II)	ARP: A2,B1,C0, mild CAA	ARP: A2,B1,C0

Scores: −, absent; +, mild; ++, moderate; +++, severe; ++++, status spongiosus. ARP defined according to Montine et al. 2012 [17] (A for amyloid-phase 0–3; B for Braak neurofibrillary stage 0–3; C for CERAD neuritic plaque score 0–3)

*SP* spongiform change, *PrP* pattern of prion protein deposition, *syn* synaptic, *AgD* argyrophilic grain disease (according to Saito et al. [28], *ARP* Alzheimer-related pathology, *CAA* cerebral amyloid angiopathy, *LB* Lewy body, staging according to Attems et al. [2], *PART* primary age-related tauopathy (according to Crary et al. [9])

*Focal

All cases showed consistent features of CJD with prominent spongiform change in cortical regions of all lobes, mainly consisting of intermediate to large size vacuoles, mostly non-confluent, partly reaching the cortico-subcortical boundary (Fig. 1a1, b1, c1, d1, e1, e3). All cases also showed a moderate superficial laminar spongiosis in frontotemporal areas, and 2 presented small focal clusters of confluent vacuoles in the occipital cortex (Fig. 1E1, e3). Moreover, 5 participants showed some ballooned neurons in the most severely affected cortical regions (Fig. 1c1).

**Fig. 2:**
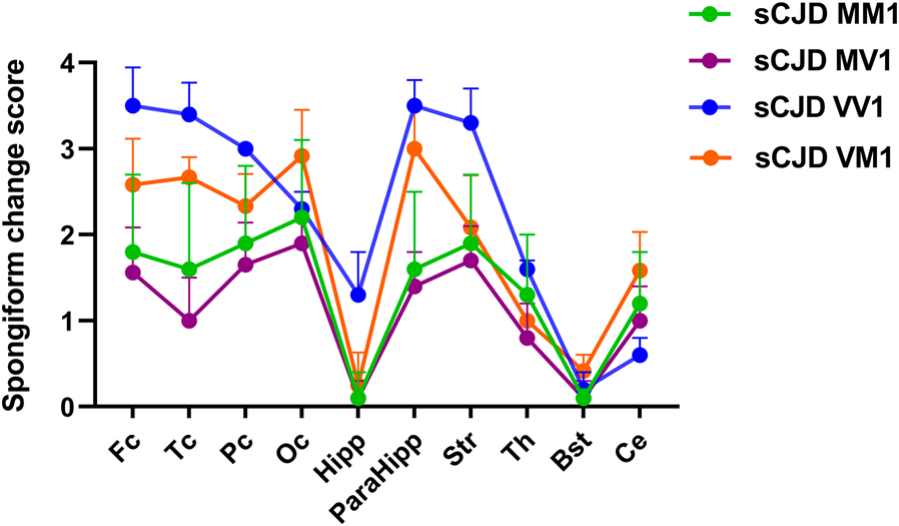
Lesion profiles of spongiform change according to sCJD subtype. Regional profiles of spongiform change severity in sCJD MM1, MV1, VM1, and VV1. In each area, the degree of spongiform change was scored as absent, 0; mild, 1; moderate, 2; severe, 3; status spongiosus 4. *Fc* frontal cortex, *Tc* Temporal cortex, *Pc* Parietal cortex, *Oc* Occipital cortex, *Hipp* hippocampus, *ParaHipp* Parahippocampal region, *Str* striatum, *Th* thalamus, *Bst* brainstem, *Ce* cerebellum

The spongiform change was also severe in the basal ganglia—caudate nucleus, accumbens, putamen - (Fig. 1B1, b1), entorhinal, and parahippocampal cortices (Fig. 1C1, c1; Table 2). Also the cingulum was significantly involved. In contrast, the amygdala, the hippocampus (CA1 sector and subiculum; Fig. 1C1) and thalamic nuclei were less involved. The alterations in the cerebellum showed a focal spongiform change in the molecular layer comprising relatively large vacuoles compared with those seen in the typical MM1 subtype (Fig. 1d1). The granular layer and the Purkinje cells were comparatively better preserved. The intensity of the overall changes was variable between the cases, but the lesioning pattern was homogeneous, with a profile somehow intermediate between the MV1 and VV1 groups. Indeed, VM1 brains showed a more prominent involvement of the occipital cortex than the other lobes like in the MV1 group but a more severe involvement of the cerebral cortex than the cerebellum as in the VV1 subtype (Fig. 2). In general, spongiform change was more prominent than the degree of neuronal loss in most areas, compared to MM1/MV1 cases, except for fronto-temporal regions, were neuronal loss was prominent. Gliosis was globally moderate and more pronounced in areas with more severe neuronal loss. Generally, PrP immunohistochemistry in the cortical areas and the basal ganglia was either negative or showed a faint synaptic pattern. We identified focal coarser PrP deposits in the occipital cortex as the only exception in two cases mainly involving the area parastriata (Brodmann area 18). In one of the patients (patient #1) DWI-MRI showed more prominent cortical hyperintensities in the affected occipital region. Focal PrP aggregates were also visible in the cerebellar molecular layer. They combined a synaptic pattern with coarser, patchy-like deposits, reminiscent but less compact than the classical "patchy-perivacuolar" pattern observed in long-duration MM(V)2C cases [20].

In a single brain (patient #5), there were also focal small plaque-like PrP deposits in substantia nigra, midbrain periaquaeductal gray, and dorsal areas of the medulla oblongata and pons. The plaque-like deposits mainly involved the gray matter, occasionally showing a perineuronal distribution and extended to the white matter.

All cases had some concomitant neurodegenerative changes, including argyrophilic grain pathology, mild tau positive neurofibrillary pathology (max. Braak stage II), and variable amounts of diffuse and cored ßA4-amyloid plaques; a single case presented limbic Lewy bodies. No TDP43 pathology was identified in any case.

### Genetic analysis

Sequencing the open reading frame of *PRNP* gene did not detect any pathogenic mutation. The codon 129 polymorphism in all cases was MV.

### PrP^Sc^ typing and characterization

**Fig. 3:**
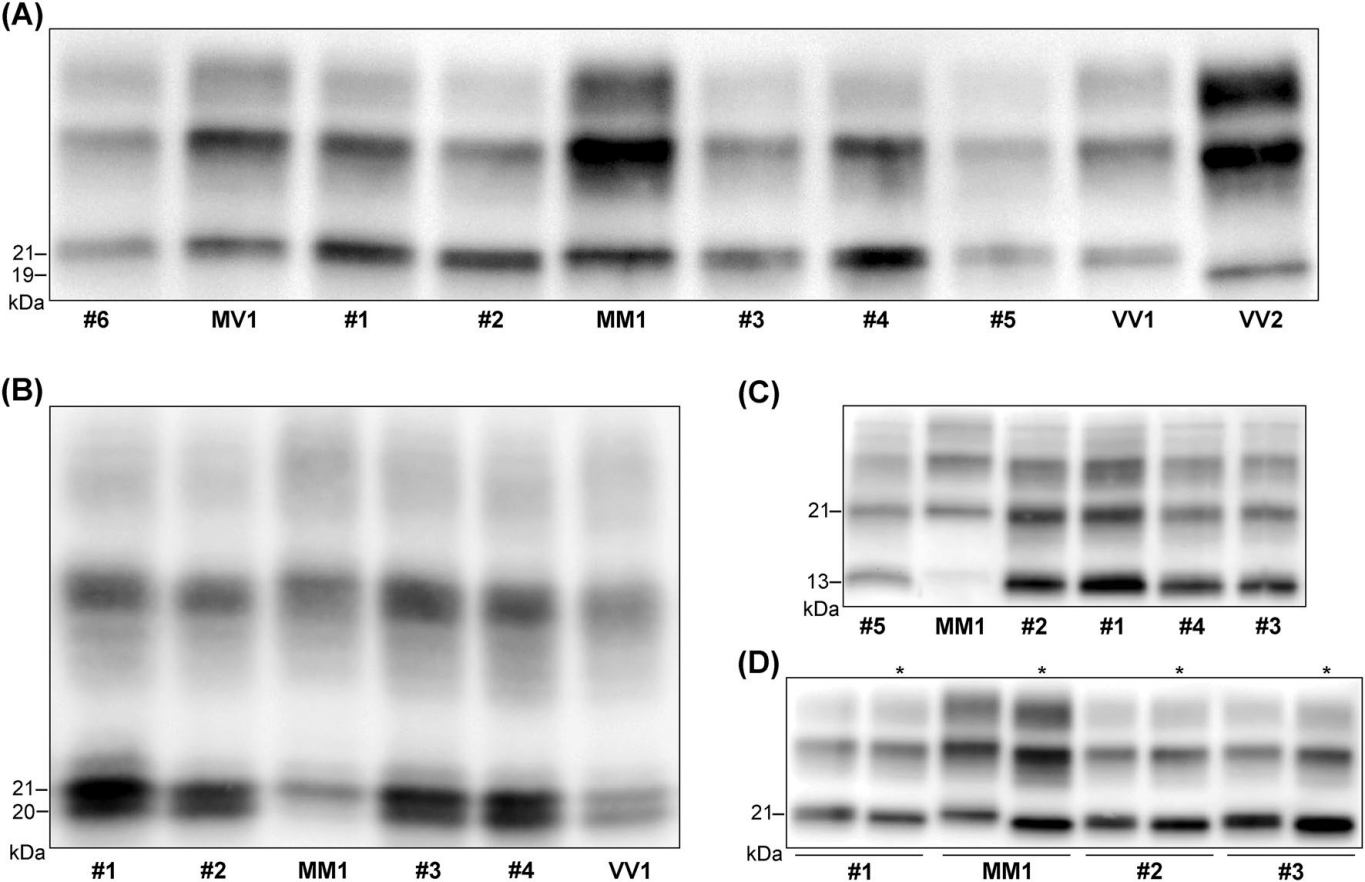
Immunoblot profiles of PK-resistant PrP^Sc^ fragments (PrP^res^) in the reported sCJD cases. **A** Immunoblot analysis of PK-treated frontal cortex homogenates by standard SDS-PAGE gel electrophoresis (running gel of 6.5 cm). The six reported cases (#1–6) are compared with sCJD cases of the MM1, MV1, VV1, and VV2 groups. Note the slightly faster migration of unglycosylated PrP^res^ in cases 1–6 and the VV1 compared to the MM1 and MV1. **B** Immunoblot analysis of PK-treated frontal cortex homogenates by high-resolution SDS-PAGE gel electrophoresis (running gel of 15 cm). A sCJD MM1 and a sCJD VV1 are included for comparison with cases #1–4. The higher resolution of the gel shows that unglycosylated PrP^res^ in VM1 and VV1 cases comprises a doublet (i.e., two bands of 21 and 20 kDa), explaining the slightly faster migration compared to MM1 and MV1 cases (they show one band of 21 kDa) **C** Immunoblot profile of PrP^res^ comprising PrP^Sc^ type 1 and the 12–13 kDa C-terminal fragments. Note the higher proportion (i.e., relative amount) of C-terminal fragments in VM1 and VV1 cases compared to MM1 and MV1 (see Table 3 for the quantitative data). **D** Comparison of the electrophoretic mobility of PK-resistant PrP^Sc^ after digestion at pH 6.9 or 8.0 (see * labels). There is a more consistent shift in migration (i.e., faster) when PK digestion is performed at pH 8 in MM1 and MV1 cases compared to VM1 and VV1. The immunoblots shown in **A**, **B**, and **C** are labeled by the N-terminal mAb 3F4. In contrast, the immunoblot in panel C is stained with the C-terminal antiserum 2301. Approximate molecular masses are in kilodaltons

**Fig. 4:**
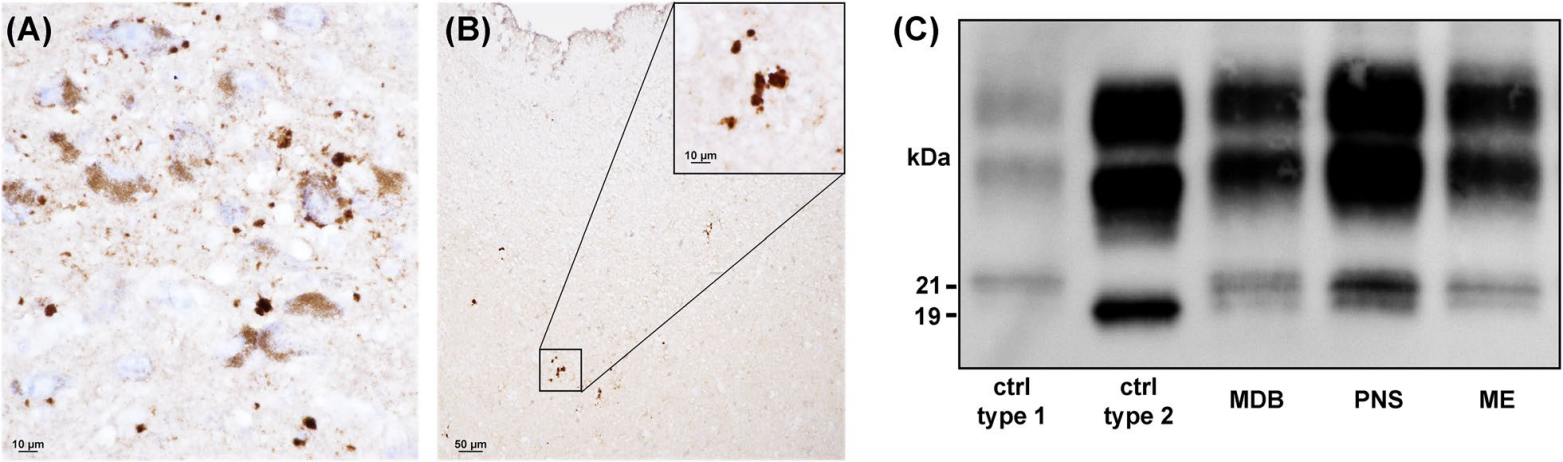
Evidence of CJD V2 features in the brainstem of patient #5. Small plaque-like PrP deposits in the substantia nigra (**A**) and midbrain periacqueductal gray (**B**) (immunohistochemistry for PrP with the mAb 3F4). **C** Western blot (mAb 3F4) shows the co-occurrence of PrP^Sc^ type 1 (unglycosylated band migrating at 21 kDa) and 2 (at 19 kDa) in multiple brainstem areas (MDB, midbrain; PNS, pons; ME medulla). Ctrl = control

Western blot analyses of PK-digested brain homogenates using the mAb 3F4 showed a PrP^Sc^ type 1 profile, irrespectively of the brain region analyzed, in 5 of the 6 cases (Fig. 3). In contrast, in patient #5 we detected the co-occurrence of PrP^Sc^ types 1 and 2 in the brainstem samples (Fig. 4), and the type 1 profile in the cerebral cortex, striatum, and cerebellum. Traces of PrP^Sc^ type 2 co-occurring with more abundant PrP^Sc^ type 1 were also present in the thalamus.

Interestingly, an in-depth comparison between the PrP^Sc^ "type 1" profile in the six identified patients and that of typical sCJD MM1 or MV1 revealed some differences. Indeed, the PrP^Sc^ extracted from the former group showed some distinctive properties previously identified in VV1 brains [18, 19]. First, the unglycosylated PrP^Sc^ fragment migrated slightly faster than the typical MM1 and MV1 subtypes (Fig. 3). We best resolved the slightly faster mobility of this band using a long gel with a higher resolution showing that the unglycosylated PK-resistant fragment in these cases comprised a doublet of 21 and 20 kDa. Secondly, PK-resistant PrP^Sc^ (PrP^res^) showed a significantly higher proportion of C-terminal truncated 12–13 kDa fragments than in MM1 and MV1 subtypes (Fig. 3, Table 3). Thirdly, as in VV1, PrP^Sc^ electrophoretic mobility after digestion at pH 6.9 and 8.0 showed a reduced migration shift (i.e., fragment migrating faster at pH 8.0) compared to the PrP^Sc^ associated with the MM1 and MV1 subtypes. Finally, the glycoform ratio showed a relative underrepresentation of the diglycosylated isoform in the cases compared to the MM1 and MV1 groups (Fig. 3, Table 3).

**Table 3:** PrP^res^ glycoform ratio and relative proportion of C-terminal (12–13 kDa) fragments in sCJD MM1, MV1, VM1 and VV1

sCJD subtype	MM1	MV1	VM1	VV1
N	18	9	6	7
D-PrP^res^ (%)	31.8 ± 2.5^a,b^	29.7 ± 2.7^c^	15.8 ± 3.9	22.1 ± 3.3
M-PrP^res^ (%)	43.6 ± 3.3^d^	43.9 ± 1.1^d^	45.4 ± 1.6	46.7 ± 1.9
U-PrP^res^ (%)	24.6 ± 3.6^a^	26.3 ± 3.2^d,e^	38.7 ± 3.5	31.2 ± 1.9
D/M-PrP^res^ ratio	1.3 ± 0.3^a,f^	1.2 ± 1.1^e^	0.4 ± 0.1	0.7 ± 0.1
U-CTF12-13 (%)*	19.3 ± 5.6^a^	22.8 ± 8.9^c^	59.3 ± 5.7	33.7 ± 9.9

Statistic comparisons (Kruskal–Wallis test): ^a^versus VM1 *p* ≤ 0.001; ^b^versus VV1 *p* ≤ 0.001; ^c^versus VM1 *p* ≤ 0.05; ^d^versus VV1 *p* ≤ 0.05; ^e^versus VM1 *p* ≤ 0.01, ^f^versus VV1 *p* ≤ 0.01. *relative to U-PrP^res^ signal

*PrP*^*res*^ protease-resistant core of type 1 (21 kDa) PrP^Sc^, *D* diglycosylated isoform, *M* monoglycosylated isoform, *U* unglycosylated isoform, *CTF12-13* C-terminal PrP^res^ fragments of 12–13 kDa

## Discussion

Here we presented clinical, neuropathological, and molecular data supporting the existence of a novel subtype of sCJD, possibly representing a previously missed prion strain/host genotype combination: the V1 strain in patients carrying MV at *PRNP* codon 129. Clinical evidence supporting this interpretation includes the relatively long disease duration (mean of 20.5 vs. 3.4 months of typical MM1/MV1 patients) (Table 4), and the absence of periodic sharp-wave complexes at EEG and early cerebellar signs, both frequent findings in MM1/MV1 patients. Moreover, most patients were male, which is also more consistent with the VV1 than the MM1/MV1 subtype [16].

**Table 4:** Comparison of demographic, histopathologic and molecular features among sCJD MM1, MV1, VV1, and VM1

sCJD subtype	MM1^#^	MV1^#^	VV1^§^	VM1
n	74*	22	7	6
Female (%)	38 (51.4)	8 (38.1)	1 (14.3)	1 (16.7)
Age at onset (years)	69.7 ± 10.2	68.8 ± 9.4	46.0 ± 10.8	74.7 ± 5.6
Disease duration (months)	3.4 ± 1.8	3.7 ± 1.8	18.0 ± 3.2	20.5 ± 6.1
*Histopathologic features*				
Vacuoles size	Small	Small	Intermediate	Intermediate
Lesion profile	Of variable severity in cerebral cortex, striatum, thalamus and cerebellum; hippocampus and amygdala spared	Virtually indistinguishable from MM1	Cerebral cortex and striatum severely affected, cerebellum relatively spared	Cerebral cortex and striatum moderately to severely affected, moderate changes in cerebellum
PrP deposits	Synaptic	Synaptic	Faint synaptic	Focally patchy in the molecular layer of cerebellum; faint synaptic elsewhere
*PrP*^*res*^* characteristics*				
WB profile of unglycosylated PrP^res^	21 kDa	21 kDa	Doublet at 21 and 20 kDa	Doublet at 21 and 20 kDa
PrP^res^ glycoform ratio	Diglycosylated > unglycosylated isoform	Diglycosylated > unglycosylated isoform	Unglycosylated > diglycosylated isoform	Unglycosylated > diglycosylated isoform
C-terminal 12–13 kDa PrP^res^ fragments**	±	±	++	++
PrP^res^ migration shift at pH 6.9 vs 8.0	Yes	Yes	Less pronounced than in MM1/MV1	Less pronounced than in MM1/MV1

Continuous variables are expressed as mean ± SD. ^**#**^sCJD MM1, MV1, and VV1 were from the ISNB cohort (2002–2021). ^§^Given the very low incidence of sCJD VV1 cases in the ISNB cohort (n = 2), we also considered the demographic features of five previously published cases [12]. *from a consecutive series between 2017 and 2019. **Relative intensity of C-terminal 12–13 kDa fragments compared to the unglycosylated PrP^res^ signal at western blot. Legend: WB, western blot

Neuropathological evidence includes the spongiform change with vacuoles of larger size than those seen in sCJD MM1/MV1 and the lesion profile with prominent cortical and striatal involvement with relative sparing of the cerebellum. Moreover, ballooned neurons also fit better with the VV1 than the MM1/MV1 sCJD subtype.

The collected molecular data align with the proposed overall interpretation by showing additional features reminiscent of the VV1 sCJD subtype [8, 18, 19]. Indeed, in these cases, the unglycosylated PrP^Sc^ fragment comprised a doublet and migrated slightly faster than the corresponding core fragment of the typical MM1/MV1 subtype [18]. Moreover, it showed a significantly higher proportion of the C-terminally truncated 12–13 kDa fragments [19]. Finally, it did not consistently demonstrate the 0.5 kDa migration shift of the unglycosylated PrP^Sc^ in MM1/MV1 subjects when performing the PrP^Sc^ PK digestion at pH 8 instead of 6.9 [18].

Of note, not all the characteristics of the sCJD cases we identified fit those previously described in the VV1 group. Indeed, the VM1 patients were significantly older and consistently showed focal coarse PrP deposits in the molecular layer of the cerebellum, occasionally also seen in the occipital cortex. Most likely, these divergent features represent a host genotype effect related to the different codon 129 genotypes (MV vs. VV). Heterogeneity of phenotypic expression of individual strains due to host genotypic variation is a well-known phenomenon in prion diseases, even within the sCJD spectrum. The most significant example is the V2 strain manifesting cerebellar kuru-type amyloid plaques and a prolonged clinical course only in individuals expressing at least one M at *PRNP* codon 129.

The finding in patient #5 of the present series deserves further comment. We believe that the case represents the first reported example of co-occurrence of V1 and V2 strains in sCJD. Indeed, previous studies identified the mixed VV1 + 2 subtype as part of the sCJD spectrum, which makes our finding of the VM1 + 2 co-occurrence not surprising [8, 20]. Given the expression of both PrP^C^ with M and V in patients heterozygous at codon 129, other combinations (e.g., VM1 + 2C) may show up. It is also worth noting that the divergent "type 2" pathology in the brainstem, also involving the substantia nigra, correlated with the clinical onset of parkinsonism in this patient.

Regarding the terminology to attribute to this novel subtype, considering the phenotypic differences outlined above with both MM1/MV1 and VV1 groups, we suggest the VM1 abbreviation to emphasize the phenotypic similarities with the V1 strain and distinguish this subtype from all the others linked to PrP^Sc^ type 1. Future studies should compare the transmission properties of the three subtypes to verify the proposed hypothesis that VM1 brains will propagate the V1 strain rather than the M1. If confirmed, it will be the time for an updated classification of sCJD comprising three subtypes linked to PrP^Sc^ type 1: the MM1/MV1, the VM1, and the VV1.

The present study results further support the central role of the polymorphic residue 129 of PrP in determining the phenotypic spectrum of human prion disease [13]. Current evidence indicates that the presence of M or V drives the possible pathological conformations that PrP^Sc^ may assume and strictly modulate their propagation [3, 26]. Indeed, up to six human strains seem to be able to propagate through PrP-129 M conversion (M1, M2C, M2T, M2-variant, M2-VPSPr, and V2), whereas only three through PrP-129 V (V1, V2, and V2-VPSPr). Moreover, except for the V2 strain, the codon 129 M represents a complete barrier to propagating the strains compatible with PrP-V and vice-versa. This scenario creates a unique situation for the MV carriers. On the one hand, they are less susceptible to the disease than the MM and VV homozygotes because they express only one compatible PrP. On the other hand, they can replicate virtually all prion strains and manifest the whole phenotypic spectrum of the disease, given that they carry both PrP-M and PrP-V. In this regard, also considering the existence of mixed phenotypes, it is still possible that other rare prion strains combinations will show up in patients carrying MV at codon 129. For example, the focal areas showing large confluent vacuoles associated with coarser PrP deposits might suggest the co-occurrence with the M2C strain. However, we could not confirm this hypothesis by demonstrating PrP^Sc^ type 2 by western blotting in these cases.

Interestingly, we only identified this phenotype in Catalonia and Italy, not Austria. Moreover, the percentage of identified cases in the screened population of definite sCJD cases was significantly higher in Catalonia than in Italy. As these cases are rare (6 among 189 screened MV cases = 3%, and a total of 1200 sCJD cases = 0.5%), it might well be that the different regional incidence is only a matter of chance rather than a geographically determined difference. Nevertheless, it will be interesting to see whether similar cases also occur in other countries and assess their incidence.

As most of the patients were > 70 years old, neuropathologic examination revealed concomitant age-related pathologies, including argyrophilic grain disease, primary age-related tauopathy in limbic regions, and a variable amount of beta-amyloid plaques [9, 12, 28]. However, it seems unlikely that these copathologies contributed significantly to some clinical features, given the florid spongiform change and CJD-specific pathology affecting the cerebral cortex and the limbic cortex, particularly the parahippocampal region and the cingulum. Indeed, in previous studies, we found that even a significant Alzheimer-related copathology has only a limited effect on the clinical manifestations of sCJD [11, 15, 27].

In conclusion, we have characterized the clinical, neuropathological, and molecular features of a novel subtype of sCJD, likely representing a missing molecular combination in the current sCJD classification, namely the V1 strain in the heterologous MV codon 129. This rare sCJD subtype mainly affects elderly patients and presents a unique neuropathological phenotype dominated by features of the V1 strain but with the association of specific PrP immunostaining features resulting from the modulatory effect of the codon 129 MV genotype.

The lack of experimental transmission data represents a significant limitation of the present study since we cannot firmly conclude that V1 prions caused the cases recognized in this study. Nevertheless, our data strongly indicate that they belong to a rare, previously unidentified sCJD subtype distinct from the typical MM1/MV1. Identifying this unique subtype in elderly patients may be relevant for the differential diagnosis of neurodegenerative dementias in the aged population and prognostic estimates in patients with the clinical diagnosis of CJD, as these patients manifest a longer disease duration compared to the typical MM1/MV1 subtype. Their early identification will also be crucial when prion protein-targeted therapies become available to test their subtype-specific effect. Finally, surveillance programs should monitor the true incidence of sCJD in the elderly, which may be higher than previously thought, especially if atypical phenotypes are increasingly registered.

## Supplementary Information

**Additional file 1: Figure S1**. Representative MR images. A1–A3: Patient #4, Spain, at 6 months of onset of symptoms. B1–B3: Patient #5, Italy, at 9 months of onset of symptoms. Diffusion weighted images of both patients reveal cortical hyperintensities, in patient 4 (A1–A3) particularly affecting the right parieto-occipital lobes, and in patient 5 (B1–B3) involving mostly the temporal, frontal, parietal and occipital lobes, predominantly of the left brain hemisphere. Basal ganglia do not show hyperintensities. A similar pattern was observed in the other patients where MRI was performed.

## Data Availability

Anonymized data are available upon reasonable request to the corresponding authors.

## Abbreviations

CJD: Creutzfeldt-Jakob disease
CSF: Cerebrospinal fluid
ISNB: Institute of Neurological Science of Bologna
LabNP: Laboratory of neuropathology
M: Methionine
mAb: Monoclonal antibody
MUW: Medical University of Vienna
PK: Proteinase K
PRNP: Prion protein gene
PrP: Prion protein
PrP^Sc^: PrP scrapie
sCJD: Sporadic CJD
V: Valine

## References

[1] Aguzzi A, Heikenwalder M, Polymenidou M (2007). Insights into prion strains and neurotoxicity. Nat Rev Mol Cell Biol.

[2] Attems J, Toledo JB, Walker L, Gelpi E, Gentleman S, Halliday G (2021). Neuropathological consensus criteria for the evaluation of Lewy pathology in post-mortem brains: a multi-centre study. Acta Neuropathol.

[3] Baiardi S, Rossi M, Capellari S, Parchi P (2019). Recent advances in the histo-molecular pathology of human prion disease. Brain Pathol.

[4] Baiardi S, Rossi M, Mammana A, Appleby BS, Barria MA, Calì I (2021). Phenotypic diversity of genetic Creutzfeldt-Jakob disease: a histo-molecular-based classification. Acta Neuropathol.

[5] Bartz JC (2021). Environmental and host factors that contribute to prion strain evolution. Acta Neuropathol.

[6] Bessen RA, Marsh RF (1994). Distinct PrP properties suggest the molecular basis of strain variation in transmissible mink encephalopathy. J Virol.

[7] Bishop MT, Will RG, Manson JC (2010). Defining sporadic Creutzfeldt-Jakob disease strains and their transmission properties. Proc Natl Acad Sci U S A.

[8] Cali I, Puoti G, Smucny J, Curtiss PM, Cracco L, Kitamoto T (2020). Co-existence of PrP^D^ types 1 and 2 in sporadic Creutzfeldt-Jakob disease of the VV subgroup: phenotypic and prion protein characteristics. Sci Rep.

[9] Crary JF, Trojanowski JQ, Schneider JA, Abisambra JF, Abner EL, Alafuzoff I (2014). Primary age-related tauopathy (PART): a common pathology associated with human aging. Acta Neuropathol.

[10] Gambetti P, Dong Z, Yuan J, Xiao X, Zheng M, Alshekhlee A (2008). A novel human disease with abnormal prion protein sensitive to protease. Ann Neurol.

[11] Grau-Rivera O, Gelpi E, Nos C, Gaig C, Ferrer I, Saiz A (2015). Clinicopathological correlations and concomitant pathologies in rapidly progressive dementia: a brain bank series. Neurodegener Dis.

[12] Knopman DS, Parisi JE, Salviati A, Floriach-Robert M, Boeve BF, Ivnik RJ (2003). Neuropathology of cognitively normal elderly. J Neuropathol Exp Neurol.

[13] Kobayashi A, Teruya K, Matsuura Y, Shirai T, Nakamura Y, Yamada M (2015). The influence of PRNP polymorphisms on human prion disease susceptibility: an update. Acta Neuropathol.

[14] Kovacs GG, Head MW, Hegyi I, Bunn TJ, Flicker H, Hainfellner JA (2002). Immunohistochemistry for the prion protein: comparison of different monoclonal antibodies in human prion disease subtypes. Brain Pathol.

[15] Kovacs GG, Rahimi J, Ströbel T, Lutz MI, Regelsberger G, Streichenberger N (2016). Tau pathology in Creutzfeldt-Jakob disease revisited. Brain Pathol.

[16] Meissner B, Westner IM, Kallenberg K, Krasnianski A, Bartl M, Varges D (2005). Sporadic Creutzfeldt-Jakob disease: clinical and diagnostic characteristics of the rare VV1 type. Neurology.

[17] Montine TJ, Phelps CH, Beach TG, Bigio EH, Cairns NJ, Dickson DW (2012). National Institute on Aging-Alzheimer's association guidelines for the neuropathologic assessment of Alzheimer's disease: a practical approach. Acta Neuropathol.

[18] Notari S, Capellari S, Giese A, Westner I, Baruzzi A, Ghetti B (2004). Effects of different experimental conditions on the PrPSc core generated by protease digestion: implications for strain typing and molecular classification of CJD. J Biol Chem.

[19] Notari S, Strammiello R, Capellari S, Giese A, Cescatti M, Grassi J (2008). Characterization of truncated forms of abnormal prion protein in Creutzfeldt-Jakob disease. J Biol Chem.

[20] Parchi P, de Boni L, Saverioni D, Cohen ML, Ferrer I, Gambetti P (2012). Consensus classification of human prion disease histotypes allows reliable identification of molecular subtypes: an inter-rater study among surveillance centres in Europe and USA. Acta Neuropathol.

[21] Parchi P, Castellani R, Capellari S, Ghetti B, Young K, Chen SG (1996). Molecular basis of phenotypic variability in sporadic Creutzfeldt-Jakob disease. Ann Neurol.

[22] Parchi P, Cescatti M, Notari S, Schulz-Schaeffer WJ, Capellari S, Giese A (2010). Agent strain variation in human prion disease: insights from a molecular and pathological review of the National Institutes of Health series of experimentally transmitted disease. Brain.

[23] Parchi P, Giese A, Capellari S, Brown P, Schulz-Schaeffer W, Windl O (1999). Classification of sporadic Creutzfeldt-Jakob disease based on molecular and phenotypic analysis of 300 subjects. Ann Neurol.

[24] Parchi P, Strammiello R, Notari S, Giese A, Langeveld JP, Ladogana A (2009). Incidence and spectrum of sporadic Creutzfeldt-Jakob disease variants with mixed phenotype and co-occurrence of PrPSc types: an updated classification. Acta Neuropathol.

[25] Parchi P, Zou W, Wang W, Brown P, Capellari S, Ghetti B (2000). Genetic influence on the structural variations of the abnormal prion protein. Proc Natl Acad Sci U S A.

[26] Rossi M, Baiardi S, Parchi P (2019). Understanding prion strains: evidence from studies of the disease forms affecting humans. Viruses.

[27] Rossi M, Kai H, Baiardi S, Bartoletti-Stella A, Carlà B, Zenesini C (2019). The characterization of AD/PART co-pathology in CJD suggests independent pathogenic mechanisms and no cross-seeding between misfolded Aβ and prion proteins. Acta Neuropathol Commun.

[28] Saito Y, Ruberu NN, Sawabe M, Arai T, Tanaka N, Kakuta Y (2004). Staging of argyrophilic grains: an age-associated tauopathy. Neuropathol Exp Neurol.

[29] Shahnawaz M, Mukherjee A, Pritzkow S, Mendez N, Rabadia P, Liu X (2020). Discriminating α-synuclein strains in Parkinson's disease and multiple system atrophy. Nature.

[30] Shi Y, Zhang W, Yang Y, Murzin AG, Falcon B, Kotecha A (2021). Structure-based classification of tauopathies. Nature.

[31] Telling GC, Parchi P, DeArmond SJ, Cortelli P, Montagna P, Gabizon R (1996). Evidence for the conformation of the pathologic isoform of the prion protein enciphering and propagating prion diversity. Science.

