# Supplementary figures and images for: Sporadic Creutzfeldt-Jakob disease VM1: phenotypic and molecular characterization of a novel subtype of human prion disease
Source: Acta Neuropathol Commun. 2022 Aug 17;10:114. doi: 10.1186/s40478-022-01415-7 (PMC9387077; doi:10.1186/s40478-022-01415-7)

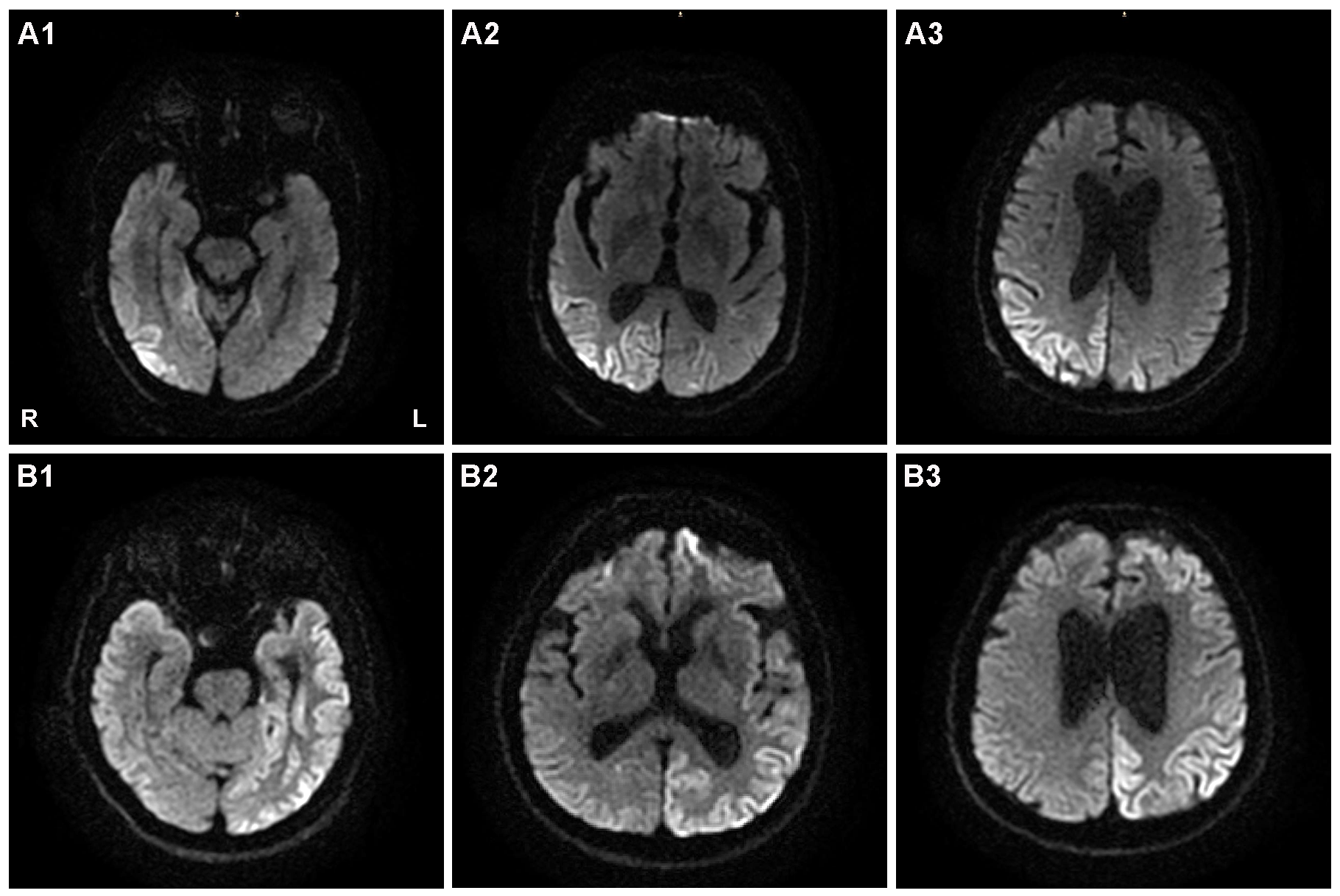

Supplement: Supplementary file 1 — Additional file 1: Figure S1. Representative MR images. A1–A3: Patient #4, Spain, at 6 months of onset of symptoms. B1–B3: Patient #5, Italy, at 9 months of onset of symptoms. Diffusion weighted images of both patients reveal cortical hyperintensities, in patient 4 (A1–A3) particularly affecting the right parieto-occipital lobes, and in patient 5 (B1–B3) involving mostly the temporal, frontal, parietal and occipital lobes, predominantly of the left brain hemisphere. Basal ganglia do not show hyperintensities. A similar pattern was observed in the other patients where MRI was performed. [file 40478_2022_1415_MOESM1_ESM.tif]
